# Perioperative Quality Initiative (POQI) consensus statement on perioperative assessment of right ventricular function

**DOI:** 10.1186/s13741-023-00351-x

**Published:** 2023-12-08

**Authors:** Stephanie O. Ibekwe, Jean Deschamps, Michael P W Grocott, Yafen Liang, Andrew Shaw, Tjorvi E. Perry, POQI IX Collaborators

**Affiliations:** 1 Department of Anesthesiology, Baylor College of Medicine, Houston, TX USA; 2 Integrated Hospital Care Institute, The Cleveland Clinic, Cleveland, OH USA; 3 Perioperative and Critical Care Theme, NIHR Southampton Biomedical Research Centre, University Hospital Southampton / University of Southampton, Southampton, UK; 4 Department of Anesthesiology, Critical Care, and Pain Medicine, University of Texas Health Science Center at Houston, Houston, TX USA; 5 Department of Anesthesiology, University of Minnesota, Minneapolis, MN USA

**Keywords:** Right ventricle, Cardiac assessment, Perioperative cardiac assessment, Perioperative cardiac screening, Right ventricle assessment

## Abstract

**Background:**

The right ventricle (RV) plays a central  role in the maintenance of effective cardiac pump function. Despite overwhelming evidence that perioperative RV dysfunction (RVD) and failure (RVF) are associated with poor clinical outcomes, there are very few published recommendations or guidelines for comprehensive, evidence-based RV assessment on the risk of developing either during the perioperative period.

**Main text:**

To address this gap, the Perioperative Quality Initiative-IX (POQI-IX) investigators group, comprised of clinical experts in anesthesiology, cardiovascular surgery, internal medicine, critical care medicine, and advanced practice nursing, has developed a consensus statement based on current literature, published society recommendations, and the clinical expertise of the group. Herein, the group provides recommendations and evidence-based tools related to perioperative RV assessment, functional screening, staging, and the clinical implications of each. These assessment tools are based on comprehensive patient evaluation consisting of physical examination, biomarker data, imaging, and hemodynamic assessment.

**Conclusion:**

This review presents a comprehensive tool for assessing perioperative RV function. We hope that this simple, intuitive tool can be applied to all phases of perioperative care and thereby improve patient outcomes.

## Background

Right ventricular (RV) function is integral to cardiac pump function through providing preload to the left ventricle and systemic circulation and maintaining interventricular interdependence (Abouzeid et al. 2017). Acute right ventricular dysfunction (RVD) may be present perioperatively, often in the setting of chronic ischemic or non-ischemic cardiomyopathies, chronic lung disease, pulmonary hypertension, or valvulopathies. For example, approximately one-third of heart failure patients with preserved left ventricular ejection fraction (LVEF) have concurrent RVD (Ahmad et al. 2021). The incidence of RVD increases to 48% when LVEF is reduced (Anavekar et al. 2007). Acute perioperative RVD can result from abrupt increases in RV afterload (e.g., acute pulmonary thromboembolic events, hypoxia, hypercapnia, acidemia) or decreased RV contractility (e.g., acute RV infarct, myocarditis, post-cardiotomy shock) (Arora et al. 2022).

A reliable and accurate method of assessment of RV function is essential for preoperative risk stratification, including when further workup is needed, how resources are allocated, identifying the appropriate care setting, and how to escalate and de-escalate intraoperative and postoperative care. Despite overwhelming evidence that perioperative RVD is associated with poor clinical outcomes, there are very few published recommendations or guidelines on which clinicians can rely to ensure comprehensive, evidence-based assessment of the risk of developing RVD during the perioperative period (Arora et al. 2022; Atkinson et al. 2017; Avendi et al. 2017; Badano et al. 2018; Barco et al. 2019; Beaubien-Souligny 2020).

To address this gap, the POQI-IX group developed a consensus statement on screening, assessing, and staging RV function during the perioperative period for patients undergoing either cardiac or non-cardiac surgery that can be applied across all phases of perioperative care using a process that has been previously described and on which the eight preceding POQI conferences have relied (Bernard et al. 2017). Briefly, the POQI-IX group comprised physicians and nurses with a broad knowledge base in the epidemiology and pathophysiology of heart failure with expertise in evaluating, caring for, and managing patients with perioperative RVD. The group included anesthesiologists, internal medicine physicians, critical care physicians, advanced heart failure and transplantation surgeons, and nurse practitioners that met in person over the course of three days. The entire group was divided into three separate groups; the authors of this manuscript were tasked with gaining consensus on how best to assess perioperative right ventricular dysfunction. To meet this objective, our smaller group of experts drafted three questions before presenting them to the entire group of experts. We used an iterative process whereby each question was discussed and modified to a final consensus question that was agreed on. The entire group of experts reached consensus on the following three questions:

1. How can we identify patients at risk of RV deterioration?
2. How can we identify RV deterioration?
3. What are the optimal modalities for assessing RV function during different phases of perioperative care?

In addressing these questions, the POQI-IX group developed a systematic approach that will be explained in the four following sections: screening of at-risk perioperative patients, perioperative staging of RV function, assessing RV function during the perioperative period, and clinical implications.

## Main text

### Perioperative RV screening tool

To facilitate early identification of patients at risk for perioperative RV deterioration, the POQI-IX group proposes the POQI-IX Individualized Right Heart Risk Assessment Tool (PIRRAT) that combines patient and surgical risk factors before surgery. The following is an account of how PIRRAT can be used in different clinical circumstances and settings. As a novel clinical tool however, PIRRAT would benefit from further testing and validation against usual standard practices in a broad sample of practice settings including inpatient, outpatient, multi-specialty, and single-specialty centers. The screening tool should be subjected to sensitivity and specificity analysis, and practicality, feasibility, and cost-effectiveness in a real clinical setting should be evaluated to ensure the tool’s suitability for use in healthcare practice. As an initial step toward developing a much-needed clinical screening tool, the POQI-IX group devised an instrument that we believe can accurately measure the clinical construct it is intended to assess based on a review of the literature and multidisciplinary expertise.

PIRRAT aims to provide an initial framework for research to validate its accuracy for identifying at-risk patients. We propose that PIRRAT be used to preoperatively screen all patients with no known advanced cardiac disease using available clinical information. In patients with elevated PIRRAT scores, additional testing such as BNP and/or TTE and optimization of RV function is recommended prior to proceeding to surgery. As a disclaimer, patients with a known history of comorbidities associated with elevated risk to RVD (e.g., severe coronary artery disease (CAD), pulmonary hypertension, congestive heart failure, and severe cardiac valvulopathies) should undergo comprehensive perioperative cardiac testing appropriate for the disease process and planned surgical procedure.

The PIRRAT scoring tool is divided into three categories: medical risk factors, surgical or procedural risk factors, and functional status (Table 1). In addition to age over 65 years, known cardiovascular and pulmonary risk factors associated with increased risk of RVD are included under patient risk factors. These include systemic hypertension, diabetes, obesity, atherosclerotic disease, acute and chronic lung disease, obstructive sleep apnea (OSA), body mass index (BMI) > 30, history of or current venous thrombotic disease, > 20 pack-year history of smoking, or the presence of acidosis or sepsis (Beaubien-Souligny 2020). Each of the medical risk factors listed in Table1 is weighted equally and, when present, is assigned a score of 1.

**Table 1:** POQI- IX Individualized Right Heart Risk Assessment Tool (PIRRAT) for early identification of patients at risk for perioperative right heart dysfunction. The total PIRRAT score is calculated by adding the medical risk score (maximum of 4) to the surgical or procedural score (maximum of 6) and then multiplying that number by the functional status score. For example, a 72-year-old patient with a BMI of 45 and sleep apnea (3 points) undergoing single lung ventilation (1 point) and functional status of 3 will have a PIRRAT score of 3 + 1 = 4 × 3 = 12—a PIRRAT score of 12 warrants a BNP measurement for additional workup and assessment

Medical risk CA	Score	Surgical or procedural risk	Score	Functional status	Score
**Age > 65 years**		Trendelenburg		1	
**Acute lung disease**		Pneumoperitoneum		2	
**Chronic lung disease**		Intracavitary		3	
**OSA**		Prone		4	
**Obesity (BMI > 30)**		Hypercapnia			
**Venous thromboembolic disease**		IVC/aortic manipulation			
**Smoking (> 20 ppy)**		Deliberate hypotension			
**Acidosis**		Sympathectomy			
**Sepsis**		Structural heart			
**CAD**^**a**^					
**Total score (max 4)**		**Total score (max 6)**		**Total score**	

*BMI* Body mass index, *OSA* Obstructive sleep apnea, *IVC* Inferior vena cava, *CAD* Coronary artery disease

^a^Medically managed CAD

Surgical or procedural approaches that increase intrabdominal and thoracic pressure, increase the risk of hypercarbia and hypoxemia, or last longer than 3 h have the potential to cause right heart strain and RVD (Avendi et al. 2017; Bootsma et al. 2022; Bootsma et al. 2022) and have therefore been included as risk factors. These include single lung ventilation, use of Trendelenburg position, laparoscopic surgery, major open procedures, major vascular surgery, neurological surgical procedures, moderate or deep sedation without an invasive airway, prone positioning, > 3-h surgical time, and bariatric surgery. Similar to patient risk factors, each surgical or procedural risk factor is weighted equally and, when present, is assigned a score of 1. Finally, the patient’s preoperative functional status at the time of assessment is based on their New York Heart Association (NYHA) Functional Status 1 through 4. It is used as a multiplier after adding the risk factors to the surgical or procedural risk factors (see Table3legend for details and explanation) (Braunwald et al. 1956).

The PIRRAT scoring system ranges from a minimum possible score of 1 to a maximum possible score of 40. In patients with elevated PIRRAT scores, additional testing and optimization of RV function are recommended during the preoperative period. For patients who score between 0 and 10, we recommend no further testing to assess the risk of RVD. For patients who score between 11 and 20, we recommend measuring BNP as the sole screening tool (Casserly and Klinger 2009; Chin et al. 2019; Chow et al. 2008). Should BNP be elevated (or if not available), we recommend TTE assessment. For patients who score over 20, we recommend measuring BNP and obtaining a TTE (Table2). If BNP and TTE are abnormal, consider referring to a specialist for invasive hemodynamic assessment. Alternate imaging assessments may include CMR, single photon emission computed tomography (SPECT), radionuclide ventriculography, PET, cardiac CT, or invasive coronary angiography (Beaubien-Souligny 2020).

**Table 2:** PIRRAT score

PIRRAT score	Recommended testing
**1–10**	No investigations
**11–20**	BNP
**> 20**	BNP + TTE

*BNP* Brain natriuretic peptides, *TTE* Transthoracic echocardiogram

In addition to the example provided in the Table 1 legend, we offer the following examples for clarity:

1. A 72-year-old long-time smoker with chronic OSA and a history of DVT who presents to the operating room for a radical neck dissection surgery has five patient risk factors (age, smoking, chronic lung disease, OSA, and venous thromboembolic disease, for a maximum of 4 points) and no surgical risk factor. With a functional status of 2, this patient’s total score is 4 + 0 = 4 × 2 = 8. With a PIRRAT score of 8, no additional assessment is warranted.
2. A 58-year-old long-time smoker with chronic obstructive pulmonary disease who presents to the operating room for a laparoscopic hysterectomy in the Trendelenburg position has two patient risk factors (smoking and chronic lung disease) and two surgical risk factors (pneumoperitoneum and Trendelenburg position). With a functional status of 4, this patient’s total score is 2 + 2 = 4 × 4 = 16. With a PIRRAT score of 16, a BNP measurement is warranted. If the institution does not provide BNP measurements, this patient should have a TTE or TEE assessment of the right ventricular function before surgery.
3. A 76-year-old obese smoker with OSA with wheezing on expiration is scheduled for laparoscopic cholecystectomy and has four patient risk factors (age, smoking, OSA, and acute lung disease; 4 points) and two surgical risk factors (pneumoperitoneum and Trendelenburg position). With a functional status of 4, this patient’s total score is 4 + 2 = 6 × 4 = 24. With a PIRRAT score > 20, a BNP measurement and a TTE or TEE assessment of the right ventricular function are warranted. If the RV function is reduced, consider referral to a specialist for hemodynamic assessment.

### RV function staging definition

**Fig. 1:**
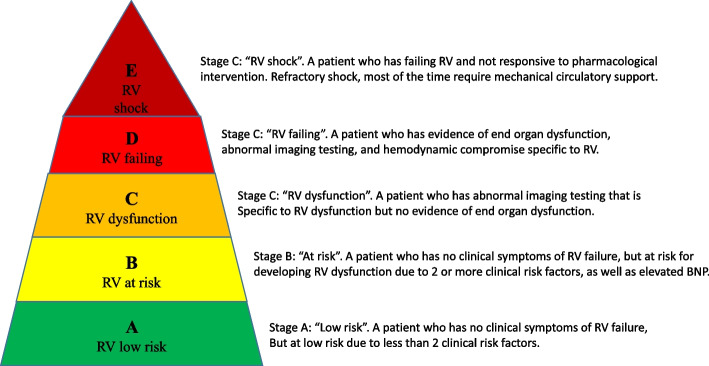
The pyramid of right ventricular function staging. RV, right ventricle

The PIRRAT scoring system described above can be used preoperatively to identify patients who have or are at risk of developing RVD and to guide further preoperative testing. Once a patient has been assigned to one of the 3 PIRRAT scoring categories (Table 2) and the testing results return, we offer a 5-stage matrix for stratifying perioperative RV function that can, in turn, facilitate resource allocation, triage of care to or from a higher-skilled facility, and intraoperative and postoperative escalation and de-escalation of care (Table 3, Fig. 1). We have intentionally kept this staging system broad to avoid pigeonholing clinicians when patients present with signs and symptoms of RV function that might fall between or across two stages. Future studies are needed to validate this staging system and assess its clinical utility.

**Table 3:** Perioperative assessment and staging of RV function from A (low risk) to E (RV shock)

Stage	Description	Physical exam	Biomarkers	Hemodynamics	Imaging
**A** **Low risk**	Fewer than two clinical or surgical risk factors but no signs or symptoms of RVD	Normal	Normal	Normal systemic blood pressure	Negative/not performed
**B** **At-risk**	Greater than two clinical or surgical risk factors but no signs or symptoms of RVD	Normal	Elevated BNP	Normal systemic blood pressure Normal CVP	Negative/not performed
**C** **RVD**	Imaging suggestive of RVD, but no evidence of end-organ dysfunction	Mild signs of RVD	Elevated BNP	Normal systemic blood pressure Normal CVP If known: Cardiac index ≥ 2.2	RV dilation reduced TAPSE, FAC, RVEF
**D** **RVF**	Evidence of end-organ dysfunction	Increased JVD, LE ascites Hepatomegaly Decreased UOP	Elevated BNP	Low systemic blood pressure requiring pharmacological interventions Cardiac index < 2.2	RV dilation, reduced TAPSE, FAC, RVEF., TR, hepatic vein congestion
**E** **RV shock**	Evidence of end-organ dysfunction unresponsive to pharmacologic intervention	Stage D criteria and altered MS, poor peripheral perfusion	Stage D criteria	Low systemic blood pressure refractory to maximal pharmacologic intervention and when mechanical circulatory support devices are in place or being considered	Stage D criteria

*BNP* B-type natriuretic peptide, *BP* Blood pressure, *CVP* Central venous pressure, *FAC* Fractional area change, *JVD* Jugular venous distension, *LE* Lower extremity, *MS* Mental status, *RVD* Right ventricle dysfunction, *RVEF* Right ventricular ejection fraction, *TAPSE* Tricuspid annular plane systolic excursion, *TR* Tricuspid regurgitation, *UOP* Urine output

#### Stage A

“Low risk” for RVD describes a patient who is not experiencing any signs or symptoms of RVD but is at risk for developing RVD during the perioperative period. Patients like this may appear well with a normal physical examination and laboratory values. However, they may have a history of chronic lung disease, high body mass index, or venous thromboembolic disease predisposing them to perioperative RVD. They may be scheduled for a high-risk procedure (e.g., a laparoscopic procedure requiring pneumoperitoneum) that can lead to increased pulmonary vascular resistance and RVD. For the comprehensive list of risk factors, please refer to Table 1. We arbitrarily categorize patients as “low risk” when they have less than two risk factors, given the high prevalence of these clinical risk factors in the general population.

#### Stage B

“At risk” for RVD describes a patient who has no clinical evidence of RVD but has two or more predisposing risk factors (from Table 1) and elevated BNP.

#### Stage C

“RVD” describes a patient who appears well and has a normal physical exam but has an elevated BNP and RV dilation on cardiac imaging.

#### Stage D

“RV Failing”: RV failure (RVF) describes a patient with signs and symptoms of heart failure and systemic hypotension. These patients may present with jugular venous distension, hepatomegaly and ascites, lower extremity edema, and reduced urine output. RVF is associated with impaired kidney function (e.g., creatinine doubling or > 50% drop in glomerular filtration rate); elevated lactate, BNP, and liver enzymes; and decreased synthetic function (elevated international normalized ratio (INR) and/or low albumin). Patients with RVF have low CO and low mixed venous oxygen saturation.

#### Stage E

“RV shock” describes a patient with RVF who is resistant to pharmacological intervention and requires mechanical circulatory support. Patients in RV shock may have altered mental status with cold, clammy, or even mottled and dusky extremities. BNP is elevated, and cardiac imaging and advanced hemodynamic assessment reveal a profoundly underperforming RV.

### RV assessment modalities

Established modalities for assessing RV function, for which normal values have been defined, are essential for reliable of diagnosis and monitoring of perioperative RVD. Understanding the roles, strengths, and limitations of each modality will enable more accurate assessment for clinical management. Each modality, including biomarkers, imaging, and hemodynamic assessment, may have multiple types of measurements that can be performed. Given a choice, the preferred measurement should always be the most consistent measurement available for each stage of care. Since the most accurate measurement is not always feasible, this group will also recommend the most commonly used measurement or an alternative that should be available in most institutions.

#### Physical exam

A thorough history and physical examination should always be the initial step in clinical diagnosis and care delivery. Jugular venous distension (JVD) and peripheral edema are non-sensitive and non-specific signs of RVD and should be used cautiously when diagnosing and treating perioperative RVD (Couture et al. 2019). Other non-specific clinical signs of RVD include shortness of breath, coughing, and wheezing, together with the manifestations of pulmonary hypertension–loud P2 on auscultation of the chest and a synchronous RV heave.

#### Biomarkers

Brain natriuretic peptides (BNP) measurement is a well-validated biomarker of LV failure, and it is a useful prognostic and risk stratification marker for RVD in patients with pulmonary hypertension. There is a paucity of data supporting its use specifically in RVD screening, but there is reasonable data supporting BNP as a screening tool for perioperative assessment of early or not previously known at-risk cardiac disease at large (Casserly and Klinger 2009; Chin et al. 2019; Dalla et al. 2019; Denault et al. 2006; Denault et al. 2013; Deschamps et al. 2023; Duceppe et al. 2017). Troponin elevation is an alternative biomarker of RVD, albeit with most literature focused on risk stratification in acute pulmonary embolism (Duceppe et al. 2020; Galie 2015; Gavazzoni et al. 2020; Giusca et al. 2010). Bilirubin can be an indirect marker of congestive RVD and, in isolation, remains non-sensitive and non-specific. As such, while there is a paucity of data specific to RVD screening, we recommend using BNP in the perioperative setting to facilitate diagnosis of RVD (Dalla et al. 2019). Most published results use N-terminal pro-BNP (Nt-ProBNP), but in the absence of comparative data between different assays, using either Nt-ProBNP or BNP is acceptable.

#### Imaging modalities

Cardiac magnetic resonance (CMR) is considered the gold standard imaging tool for assessing RV function against which other modalities should be compared (Gonzalez et al. 2003; Guazzi et al. 2013; Hamilton-Craig et al. 2016). When CMR is unavailable or does not comply with the patient’s stage of care, we recommend using a transthoracic echocardiogram (TTE). In the operating room or when access to the patient’s chest is not immediately available, a transesophageal echocardiogram (TEE) can be used (Guazzi et al. 2013).

RV ejection fraction (RVEF) is the most consistent imaging measurement of systolic RV function, with the strongest prognostic value regardless of modality (Badano et al. 2018; Hamilton-Craig et al. 2016; Hardegree et al. 2013; Heidenreich et al. 2022). CMR-derived RVEF is preferred to 3D echocardiography-derived RVEF, as 3D echocardiography tends to systematically underestimate volumes and overestimated RVEF (Henzler et al. 2012; Humbert 2022; Iglesias-Garriz et al. 2012; Jessen et al. 2022). These two modalities require time and expertise to perform, limiting their routine use (Kind et al. 2010; Kind et al. 2011; Knight et al. 2015). CMR is not accessible intra-operatively and may be limited by the inability to tolerate rate-modifying agents or perform breath holds. 3D RVEF may be limited by an inability to obtain adequate image quality with TTE or an inability to obtain a preoperative TEE due to the patient’s clinical condition or an intraoperative TEE due to surgical conditions. A 2D echocardiography-derived RVEF by Biplane Simpson’s is not recommended (Konstam et al. 2018).

While RVEF may be the most consistent parameter for assessing RV function, the most commonly used imaging measurement is fractional area change (FAC). FAC is a global assessment that incorporates radial and longitudinal components of RV contraction. It outperforms unidirectional measures of RV function and correlates more closely with CMR when compared to peak RV systolic myocardial velocity (S’) and tricuspid annular planar systolic excursion (TAPSE). TAPSE and S’ are reasonable alternatives as they are easy to perform and can identify patients with abnormal RVD within limitations (Guazzi et al. 2013; Hamilton-Craig et al. 2016; Hardegree et al. 2013; Konstam et al. 2018; Kovacs et al. 2019; Lange et al. 1989; Lankhaar et al. 2006; Lee et al. 2018; Lega et al. 2009). While easy to perform, TAPSE and S' are often abnormal after cardiac surgery; current evidence suggests that many pathological states result in abnormal longitudinal function despite preserved RVEF through increased radial and anteroposterior contraction which forms the basis of our recommendation (Hamilton-Craig et al. 2016; Knight et al. 2015; Konstam et al. 2018; Lewis et al. 2020). RV myocardial performance index (RVMPI) is an accurate metric but is more technically difficult to perform and often pseudo-normalizes in the setting of increased right atrial pressure (RAP) and is highly dependent on the operator (Gonzalez et al. 2003; Hamilton-Craig et al. 2016; Konstam et al. 2018). A new hemodynamic marker (TAPSE/PASP) combines TAPSE, a measure of contractility and pulmonary artery systolic pressure (PASP) and a measure of pressure. TAPSE/PASP correlates well with invasive measurements of RV-PA coupling but remains unvalidated for outcomes in large-scale studies (López-Candales et al. 2007; Mariano-Goulart et al. 2003). The inferior vena cava diameter and collapsibility with respiration can be used to estimate RAP as a marker of volume status. Hepatic vein flow reversal indicates severe TR, whereas leftward interatrial septal bulging indicates RAP or volume overload (Table4).

**Table 4:** Echocardiographic parameters and values to assess RV function

Variable	Imaging type	Calculation	Thresholds associated with clinical events in specific populations
**RVEF**	CMR 3D TTE/TEE	(RVEDV-RVESV)/RVEDV	Abnormal < 44%
**FAC**	2D TTE/TEE	(RVEDA-RVESA)/RVEDA	Abnormal < 35%
**TAPSE**	M-Mode	Maximal TA displacement	Abnormal < 16 mm
**S'**	TDI	Maximal systolic velocity of tricuspid annulus	Abnormal ≤ 9.6 cm/s
**RVMPI**	PWD TDI	(TCOT-RVET)/RVET	PWD RVMPI abnormal > 0.40 TDI RVMPI abnormal > 0.55
**PASP**	CWD	PASP = 4 * TRVmax^2^	Abnormal > 36 mmHg
**mPAP**	CWD	mPAP = (0.61 × PASP) + 2 mPAP = 4 x (PRVmax v)2 + RAP mPAP = 90—(0.62 × RVOT AT) mPAP = TR jet VTI + RAP	Abnormal > 25 mmHg
**TAPSE/PASP**	M-Mode CWD	Maximal TA displacement/4 * TRVmax^2^	Abnormal < 0.36 mm/mmHg
**RV basal dimension**	2D TTE/TEE	Maximal dimension at TA in focused RV view	Abnormal > 41 mm Abnormal > 21 mm^2^/m^2^
**RVEDA**	2D TTE/TEE	End-diastolic RV endocardial border area tracing	Abnormal men > 126 mm^2^/m^2^ Abnormal women > 115 mm^2^/m^2^
**RVEDV**	CMR 3D TTE/TEE	End-diastolic RV endocardial border volume tracing	Abnormal men > 87 ml/m^2^ Abnormal women > 74 ml/m^2^
**Strain**	2D TTE/TEE 3D TTE/TEE	Speckle-tracking strain imaging on RV	Abnormal RVFLS ≤ − 19% Abnormal RVGLS ≤ − 17%

*TTE* Transthoracic echocardiogram, *TEE* Transesophageal echocardiogram, *PWD* Pulsed wave Doppler, *CWD* Continuous wave Doppler, *CMR* Cardiac magnetic resonance, *RVEDV* Right ventricle end-diastolic volume, *RVESV* Right ventricle end-systolic volume, *RVEDA* Right ventricle end-diastolic area, *RVESA* Right ventricle end-systolic area, *TAPSE* Tricuspid annulus planar systolic excursion, *TA* Tricuspid annulus, *TDI* Tissue-Doppler imaging, *RVMPI* Right ventricle myocardial performance index, *TCOT* Tricuspid closure to opening time, *RAP* Right atrial pressure, *AT* Acceleration time, *TRV* Tricuspid regurgitation velocity, *PRV* Pulmonary regurgitation velocity, *RVFLS* RV Free wall longitudinal strain, *RVGLS* RV Global longitudinal strain

#### Hemodynamic modalities

Right heart catheterization using a pulmonary artery catheter (PAC) provides a range of essential hemodynamic measures including RA or central venous pressure (CVP), pulmonary artery pressure (PAP), pulmonary capillary wedge pressure (PCWP) as a surrogate for left ventricular end-diastolic pressure, RV cardiac output (CO), and mixed venous oxygen saturation. In the setting of established RVD going for an at-risk surgery, we recommend using a PAC for measuring CVP, PAP, PCWP, and CO. This is concordant with recommendations of major societies (Melenovsky et al. 2014; Miller et al. 2016). While there are established risks to PAC insertion that include arrhythmias and rare events of RV or pulmonary artery perforation, it is our view that the benefits of early detection of clinical worsening and monitoring of response to management offset the risks. When expertise is available, clinicians have the option of using an RV-port PAC to assess RVP (Mirambeaux et al. 2020; Moceri et al. 2018; Nagata et al. 2017).

Despite being the most consistent measure of CO, direct Fick-derived CO with gas rebreathing is not practical in the operating room or intensive care setting and is rarely available (Narang et al. 2022; New 1979; Opotowsky et al. 2017). The best readily available alternative is thermodilution-derived CO, as it can be easily performed in the perioperative setting and is consistently more predictive of mortality in large cohorts when compared with indirect or estimated Fick. Continuous or intermittent thermodilution cardiac output can be measured depending on the PAC used (Otani et al. 2020; Pearse et al. 2014; Pereira et al. 2020). When thermodilution-derived CO is unavailable, echocardiography-derived CO has demonstrated reasonable accuracy in critically ill patients in multiple disease states compared to invasive measurements (Rajagopal et al. 2023). TTE and TEE thus remain an alternative to thermodilution-derived CO in cases when PAC cannot be used. Pulmonary artery pulsatility index (PAPi) and the ratio of mean arterial pressure to mean pulmonary arterial pressure (MAP: mPAP) are accurate indices of RVD, especially when used as a trend. Both indices have been validated in several patient populations as valid markers of RV function, particularly in cardiac surgery and ventricular assist device settings (Table5) (Ramakrishna et al. 2005; Raymond et al. 2019; Robitaille et al. 2006; Rong et al. 2020).

**Table 5:** Hemodynamic assessment of RV function

Variable	Calculation	Thresholds associated with clinical events in specific populations
**RAP**	RAP (or CVP)	> 15 mm Hg (RHF after LVAD)
**Right-to-left discordance of filling pressures**	RAP:PCWP	> 0.63 (RHF after LVAD); > 0.86 (RHF in acute MI)
**PA pulsatility index**	(PASP − PADP)/RAP	< 1.0 (RHF in acute MI); < 1.85 (RHF after LVAD)
**RV stroke work index**	(MPAP − CVP) × SVI	< 0.25–0.30 mm Hg·L/m^2^ (RHF after LVAD)
**PVR**	(MPAP − PCWP)/CO	> 3.6 WU (RHF after LVAD)
**PA compliance**	SV/(PASP − PADP)	< 2.5 mL/mm Hg (RHF in chronic HF, RV-PA coupling in PAH)
**CO**	Thermodilution	CO < 4.2L/min
**CI**	Fick’s calculation	CI < 2.4 L/min/m^2^

*CO* Cardiac output, *CI* Cardiac index, *CVP* Central venous pressure, *LVAD* Left ventricular assist device, *MI* Myocardial infarction, *MPAP* Mean pulmonary artery pressure, *PA* Pulmonary artery, *PADP* Pulmonary artery diastolic pressure, *PAH* Pulmonary artery hypertension, *PASP* Pulmonary artery systolic pressure, *PCWP* Pulmonary capillary wedge pressure, *PVR* Pulmonary vascular resistance, *RAP* Right atrial pressure, *RH* Right heart, *RHF* Right heart failure, *RV* Right ventricular, *SV* Stroke volume, *SVI* Stroke volume index (SVI = stroke volume/BSA), *WU*Woods units. Adapted with permission from Kapur et al. (Otani et al. 2020) Copyright © 2017, American Heart Association

We do not currently recommend the use of non-invasive arterial tracing analysis for the measurement of RV function. While extensive data exists in terms of accuracy and goal-directed therapy in perioperative settings for non-cardiac surgery (Roth et al. 2018; Rudski et al. 2010), there is no data specifically for RVD, and there are concerns of systematic overestimation of CO in HF patients (Shimada et al. 2010). Stroke volume variability, which forms the basis for goal-directed management using these devices, may be more reflective of cardiopulmonary interactions than hypovolemia in RV dysfunction and lead to inappropriate management.

#### Areas of innovation

Emerging literature suggests that echocardiography-derived 2D RV free-wall longitudinal strain may be an early measure of RVD, outperforming 3D RVEF, FAC, and other markers of longitudinal RV function (Souza et al. 2007; Subramani et al. 2022; Surkova et al. 2022; Tadic et al. 2021; Tadic 2017; Tamborini et al. 2008). Technically easier to perform than 3D RVEF, RV strain analysis does not require 3D acquisition and is considered the preferred method for early identification of perioperative RVD (Dalla et al. 2019; Guazzi et al. 2013; Surkova et al. 2022; Tadic et al. 2021; Tadic 2017; Tello et al. 2019; Tokodi et al. 2021; Vizzardi et al. 2015). Venous excess ultrasound (VExUS) uses Doppler technology in the hepatic, portal, and intrarenal veins to identify patterns of flow abnormality reflective of increased right heart pressures but only has limited clinical outcome data in cardiac surgery, heart failure, and general ICU populations (Wiese 2000; Zaidi et al. 2020). While these are exciting advances, there is currently insufficient evidence to recommend RV strain analysis and VExUS technology to diagnose and treat RVD in the perioperative setting.

### Clinical implications

**Fig. 2:**
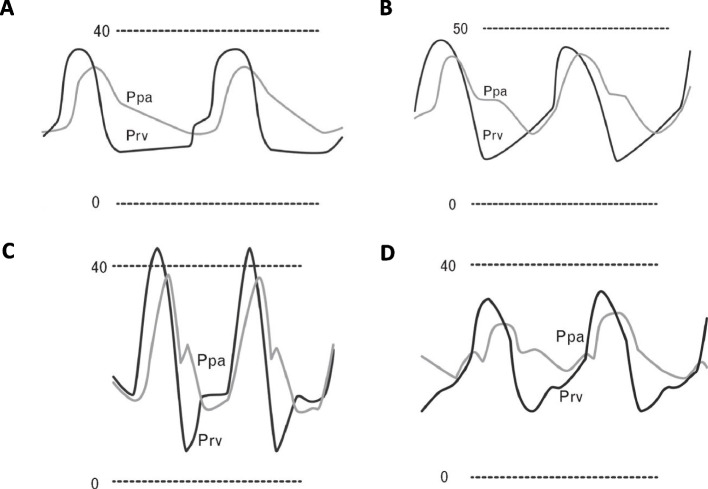
Continuous right ventricular (RV) pressure monitoring showing different stages of right ventricular dysfunction. This figure shows the simultaneous monitoring and overlap of RV and pulmonary artery (PA) pressure waveforms. The normal RV diastolic pressure slope is horizontal due to its high compliance (**A**). This slope will change from horizontal to oblique slope during RV dysfunction (**B**). The slope will further develop into a square root shape during RV failure (**C**). With severe RV failure, the RV systolic upstroke delays, and RV pulse pressure decreases (**D**). Adopted from Denault et al. (Avendi et al. 2017) with permission

RVF is one of the most challenging clinical situations to manage. Therefore, a fundamental question in caring for patients at risk of perioperative RVD is: “Are there early or predictive signs of RV deterioration from one stage to the next (e.g., from RVD to RVF or from RVF to RV shock)?” Echocardiographically, early signs of RVF include progressive RV dilation and interatrial leftward septal bulging. In addition, continuous monitoring and assessment of right heart waveform morphology are emerging as a potentially useful way to predict RVD. Moreover, continuous monitoring and assessment of right heart waveform morphology is emerging as a potentially useful way to predict RVD. For example, right atrial A wave equal to or greater than V wave indicates RVD, while A wave less than V wave and a pronounced Y-descent indicates RVF. Continuous right ventricular pressure waveform monitoring using an Oximetry Swan Ganz Paceport catheter (Edwards Lifesciences Corp, Irvine, CA, USA) is another emerging technique to detect early signs of deteriorating RV function (Atkinson et al. 2017; Zhang et al. 2019). The RV diastolic slope is typically horizontal or slightly increasing in a normal right ventricle due to normal RV compliance (Fig.2A). As RV function continues to deteriorate from RVD to RVF, the diastolic slope will increase (Fig. 2B) until it looks like a square root symbol (Fig. 2C). As RVF worsens, the systolic upstroke is delayed, and the right ventricular pulse pressure is reduced (Fig. 2D). The clinical utility and prognostic value of continuous RV waveform monitoring is yet to be confirmed in large-scale studies across different patient populations.

Importantly, no parameter in isolation should be used to identify clinically significant RV failure. The assessment of RV function requires a multimodal approach with careful evaluation of trends across clinical, laboratory, hemodynamic, and imaging parameters.

## Conclusion

Perioperative RVD is associated with significant morbidity and mortality. There are comorbidities and perioperative events that can predispose patients to RVD and RVF. In this review, we propose the POQI-IX Individualized Right Heart Risk Assessment Tool (PIRRAT) and outline how this may synthesize patient and surgical risk factors to guide preoperative workup before surgery. We have also drafted an RV function staging system to aid perioperative risk stratification, resource allocation, and when to escalate care. In creating these tools, we hope to provide a simple yet comprehensive system that may permit busy clinicians to organize their approach to the assessment and management of right heart dysfunction in the perioperative setting. Before adoption, both tools will need validation. We hope that these tools eventually lead to improved clinical care delivery and patient outcomes.

## Data Availability

Not applicable.

## Abbreviations

AT: Acceleration Time
BMI: Body mass index
BNP: B-Type natriuretic peptide
BP: Blood pressure
CAD: Coronary artery disease
CMR: Cardiac magnetic resonance
CO: Cardiac output
CVP: Central venous pressure
CW: CW Doppler
FAC: Fractional area change
INR: International normalized ratio
IVC: Inferior vena cava
JVD: Jugular venous distension
LE: Lower extremity
LV: Left ventricle
LVAD: Left ventricular assist device
LVEF: Left ventricular ejection fraction
MAP: Mean arterial pressure
MPAP: Mean pulmonary artery pressure
MS: Mental status
NT-proBNP: N-terminal pro-BNP
NYHA: New York Heart Association
OSA: Obstructive sleep apnea
PA: Pulmonary artery
PAC: Pulmonary artery catheter
PADP: Pulmonary artery diastolic pressure
PAH: Pulmonary artery hypertension
PAP: Pulmonary artery pressure
PAPi: Pulmonary artery pulsatility index
PASP: Pulmonary artery systolic pressure
PCWP: Pulmonary capillary wedge pressure
PIRRAT: POQI IX Individualized Right Heart Risk Assessment Tool
POQI-IX: Perioperative Quality Initiative- IX
PRV: Pulmonary Regurgitation Velocity
PVR: Pulmonary vascular resistance
PW: Pulsed Wave Doppler
RAP: Right Atrial Pressure
RH: Right heart
RHF: Right heart failure
RV: Right ventricle (ventricular)
RVD: Right ventricular dysfunction
RVEDA: Right ventricle end-diastolic area
RVEDV: Right ventricle end-diastolic volume
RVEF: Right ventricular ejection fraction
RVESV: Right ventricle end-systolic volume
RVF: Right ventricular failure
RVFLS: RV Free Wall Longitudinal Strain
RVGLS: RV Global Longitudinal Strain
RVMPI: RV myocardial performance index
S': Systolic myocardial velocity
SPCT: Single photon emission computed tomography
SV: Stroke volume
SVI: Stroke volume index
TA: Tricuspid annulus
TAPSE: Tricuspid annular plane systolic excursion
TCOT: Tricuspid Closure to Opening Time
TDI: Tissue-Doppler Imaging
TEE: Transesophageal echocardiogram
TR: Tricuspid regurgitation
TRV: Tricuspid Regurgitation Velocity
TTE: Transthoracic echocardiogram
UOP: Urine Output
VExUS: Venous excess ultrasound
WU: Woods units
