# Correction: Perioperative Quality Initiative (POQI) consensus statement on perioperative assessment of right ventricular function

**DOI:** 10.1186/s13741-025-00609-6

**Published:** 2025-11-28

**Authors:** Stephanie O. Ibekwe, Jean Deschamps, Michael P. W. Grocott, Yafen Liang, Andrew Shaw, Tjorvi E. Perry, POQI IX Collaborators

**Affiliations:** 1 Department of Anesthesiology, Baylor College of Medicine, Houston, TX USA; 2 Integrated Hospital Care Institute, The Cleveland Clinic, Cleveland, OH USA; 3 Perioperative and Critical Care Theme, NIHR Southampton Biomedical Research Centre, University Hospital Southampton/University of Southampton, Southampton, UK; 4 Department of Anesthesiology, Critical Care, and Pain Medicine, University of Texas Health Science Center at Houston, Houston, TX USA; 5 Department of Anesthesiology, University of Minnesota, Minneapolis, MN USA

**Correction** **:** **Perioper Med 12, 66 (2023)**

**https://doi.org/10.1186/s13741-023–00351-x**

Following the publication of the original article (Ibekwe, et al. 2023), the authors identified an error in the Competing interests section. The statement “Mike Grocott is the Editor-in-Chief of the Perioperative Medicine” should be added to the section. The section should read:

**Competing interests**

TEP has consulted for Edwards LifeSciences in the past and currently receives funding from Edwards LifeSciences to study right heart dysfunction in cardiac surgical patients. UMN NCT04478890. MG has served as a consultant for, and received unrestricted research funding from, Edwards Lifesciences. Mike Grocott is the Editor-in-Chief of the Perioperative Medicine.

The Competing interests section has been updated above and the original article (Ibekwe, et al. 2023) has been corrected.
